# Supplementary material for: The K/HDEL receptor does not recycle but instead acts as a Golgi-gatekeeper
Source: Nat Commun. 2023 Mar 23;14:1612. doi: 10.1038/s41467-023-37056-0 (PMC10036638; doi:10.1038/s41467-023-37056-0)

Supplementary Figure 1: construction of a 5'-YFP-TM domain Pp*ERD2B-1* fusion locus

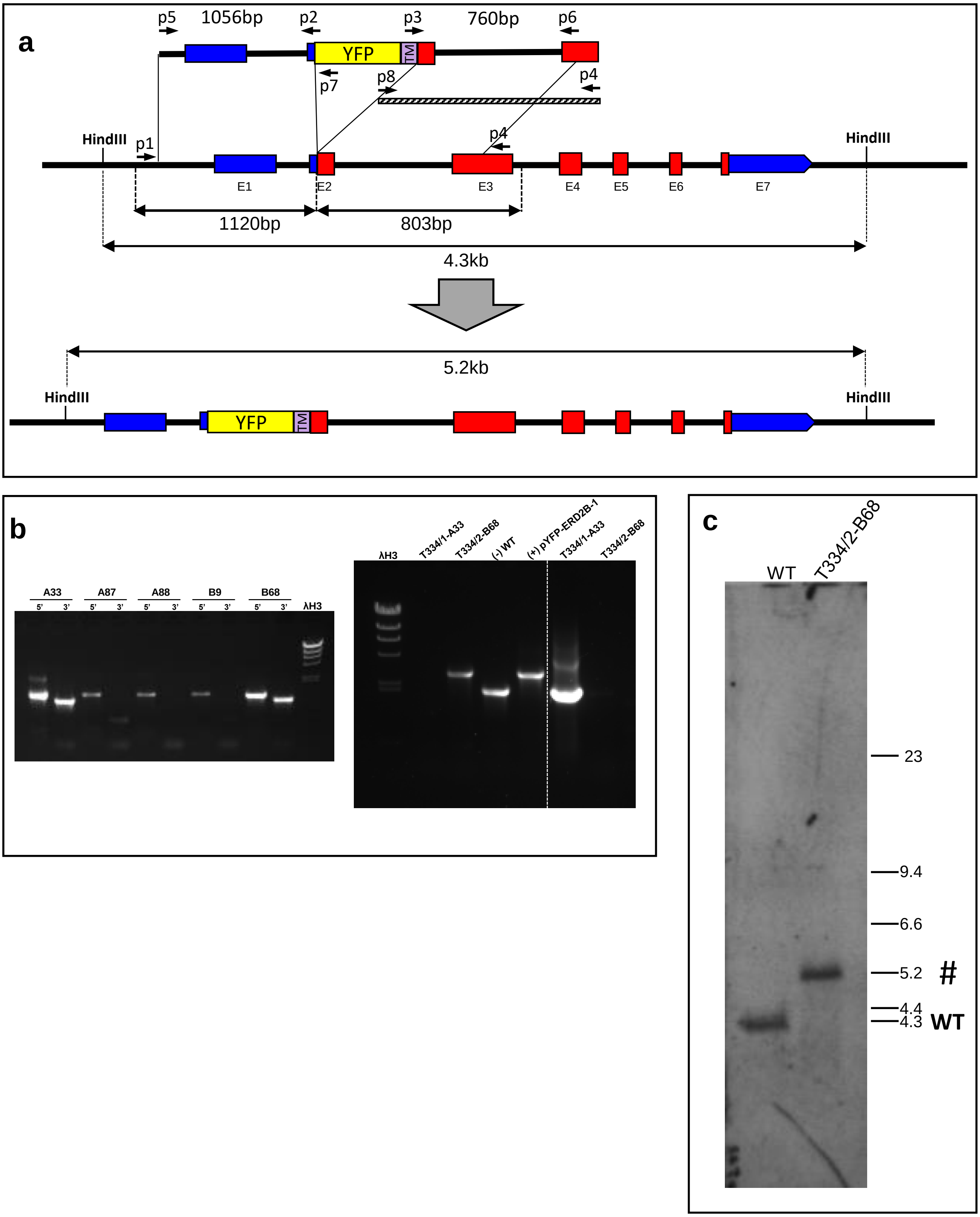

Supplementary Figure 1: Construction of a 5’-YFP-TM domain PpERD2B-1 fusion locus.

a Overview of gene-targeted knock-in strategy. The YFP-TM-ERD2B-1 fusion construct was assembled by overlap PCR and cloned in a plasmid vector. The targeting fragment was PCR-amplified using primers p1/p6 and used to transform *P. patens* protoplasts together with a circular plasmid carrying an NPTII selection cassette.

b Plants regenerating on medium containing G418 were analysed by PCR for targeted integration of the YFP-fusion sequence, using primers p1/p7 to detect 5’-targeting and p3/p4 to detect 3’-targeting (left panel). Size markers in this and subsequent images are fragments derived by digestion of λ DNA with HindIII (λH3). The two lines in which correct targeting was observed were further analysed for the presence of a single copy of the knock-in sequence (right panel), using the external primers p1/p4, to the left of the dashed line, and for the presence of concatenated transgenic replacements using primers p2/p3, to the right of the dashed line.

c A single line (T334/2B-68) in which a single copy knock-in was detected was further analysed by Southern blotting to confirm the absence of off-target integration events. Genomic DNA was digested with HindIII and the blot probed with a fragment amplified for digoxigenin labelling using primers p4/p8 corresponding to the YFP-TM-cds exons 1 and 2. A single fragment corresponding to the expected HindIII fragment of 5.2 kb was detected (highlighted by #). The WT fragment size is 4.3kb (WT).

Supplementary Figure 2: construction of Pp*ERD2B-2Δ* deletion mutant

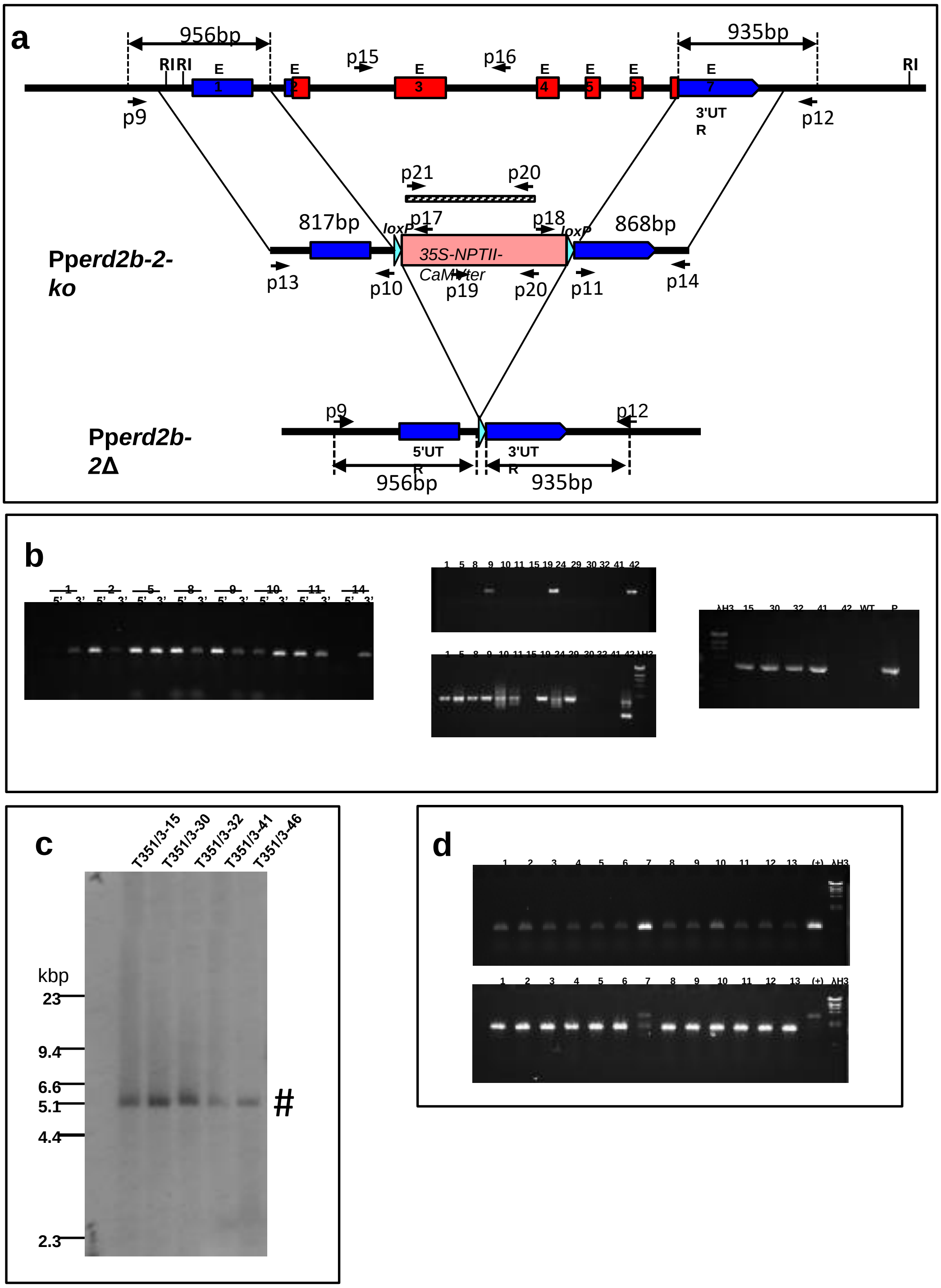

Supplementary Figure 2: Construction of PpERD2B-2Δ deletion mutant

a Overview of targeted deletion strategy. 5'- and 3'- targeting fragments were PCR-amplified from genomic DNA using primers p9/p10 and p11/p12, generating fragments of 956bp and 935bp, respectively. These were cloned on either side of the NPTII selection cassette in the Ecl136I and EcoRV sites of the vector pMBL5DLΔSN, in which the selection cassette is flanked by loxP sites (blue triangles) generating plasmid pERD2B-2KO. Amplification of the cloned fragment with primers p13/p14 generated a targeting fragment that was delivered into protoplasts to generate gene-targeted lines (Pperd2b-2-ko). Following verification of single-copy targeted transformants, the NPTII selection cassette was then deleted by transient expression of a Cre recombinase to generate the Pperd2b-2Δ mutant line.

b Verification of gene targeted Pperd2b-2-ko lines was by PCR amplification with the primers p9/p17 and p12/p18 to confirm correct targeting at the 5'- and 3'- ends of the PpERD2B-2 gene, respectively (left panel). Lines retaining a wild-type locus were identified by amplification with primers p15/p16 and excluded from further analysis (centre panel, top), and lines in which the targeting cassette had integrated as a concatemer were similarly excluded, following identification by amplification with primers p10/p11 (centre panel, bottom). Integration of a single copy at the targeted locus was confirmed by PCR using the external gene-specific primers p9/p12 (right panel). Track 'P' shows amplification of the transgene from the plasmid pERD2B-1-KO.

c Genomic DNA from lines in which the WT locus had been replaced by the selection cassette was digested with EcoRI for analysis by Southern blotting, using the 35S promoter-NPTII sequence (amplified for digoxigenin labelling with primers p20/p21) as a hybridisation probe. This identified 5 lines containing the expected EcoRI fragment (5.1kbp), indicated by #.

d Cre-mediated deletion of selection cassette. Line T351/3-15 was selected for transfection with a plasmid containing the Cre recombinase gene, for transient expression, in order to delete the selection cassette. Following a selection cycle, G418-sensitive plants were analysed by PCR amplification with primers p19/p20 (top panel) to identify plants that retained a selection cassette, and with primers p9/p12 to verify its deletion (lower panel). Of the 13 regenerating lines analysed, only line 7 retained a copy of the selection cassette (the track (+) represents a control amplification of the undeleted line T351/3-15).

Source data guidance for Supplementary Figures 1 and 2

Further information about the Lambda DNA Hind III size marker

Lambda HindIII is a standard molecular weight marker providing bands of 23.1 , 9.4, 6.6, 4.3, 2.3, 2.0 and 0,56 in kb. It is included in Suppl. Figures 1b, 1c, 2b, 2c, 2d. In the case of Suppl. Figures 1c and 2c, the characteristic bands expected from the Southern blot are also indicated in bp.

Knock-in of YFP to PpERD2B-1

Transformations: T334/1 and T334/2 using a markerless strategy, and screening explants by high-throughput “green PCR”.

We started off checking 95 plants from the two transformations for targeting at the 5’- end. However, this proved problematic, since nearly all the lines contained a false-positive amplicon.

T334/1 (gel yk180307-1 and -2) 95 plants analysed for each of 2 plates (A and B)

T334/2 (gel yk180308-1 and -2) 95 plants analysed for each of 2 plates (A and B)

So, the same plants were analysed for 3’-targeting events, with an increased number of plants from each transformation

T334/1 (gels yk180309-1 and -2) 95 plants (Plate A)

T334/2 (gels 180309-4 and -5) 95 plants (Plate B)

Plus additional T334/1 with 95 plants from 2 more plates (Plate C Gels yk180313-2and -3; Plate E Gels yk180314-3 and 4;) and 2 more plates of T334/2 (Plate D Gels yk100314-1 and 2; Plate F Gels yk180314-5 and -6)

A total of 760 plants

From these, 9 plants that generated a convincing 3’-targeting PCR product were selected for further analysis based on the extraction of DNA by the CTAB method (rather than the “green PCR” crude homogenates used in first-pass screening) for 5’ and 3’-targeting

(Gel yk180411-2: T334/1-A33, -A87, -A88, T334/2-B9, T334/2-B68, T334/2-D94, T334/2-F5, T334/2-F16 and T334/2-F37) – plants A33 and B68, were identified as both-end targeted.

These plants were then tested using external gene-specific primers to determine whether they contained a single copy of the knock-in sequence, and using transgene-specific outward-pointing primers to identify concatenated integrants.

(Gel yk180417-1) – This identified a single plant in which a single copy of the integrated sequence was present in the ERD2B1 locus: T334/2-B68

A Southern blot confirmed that there was no additional off-target insertion of the transgene elsewhere in the genome (2018-05-25 hyb) (Note that this image additionally shows analysis of other lines unrelated to this paper, and thus cropped from the image provided for this MS).

This plant was used for the subsequent second transformation to delete the ERD2B-2 gene.

ERD2B-2 deletion

Unlike the targeted knock-in transformation – which necessarily used a transforming fragment that lacked a selection marker gene, the construction of the knockout used an antibiotic resistance selection cassette replacing the ERD2B-2 sequence in order to select transformed plants. The selection of plants known to have taken up the transforming fragment meant that many fewer plants required to be screened by PCRs

In this experiment three transformations were carried out: numbers T351/1, T351/2 and T351/3.

First, 47 transformants were tested by green PCR to see if they retained a wild-type copy of the ERD2B-2 gene. 22 plants did contain a WT sequence, and so were discarded from further analysis. (gel yk180716-3).

16 of the remaining plants were tested by green PCR for 5' and 3'- targeting (gel yk180717-1). Supplementary figure 2b contains an image of the first row of tracks from this gel (plants 1-14) as the left-hand image.

Plants identified as probably targeted at either end were then propagated for isolation of good-quality DNA by the CTAB method and re-screened for the presence of a WT locus (Supplementary Figure 1b, central upper panel). The source gel image is gel yk180727-3 (upper panel) showing that lines 9 and 24 retained a WT locus undetected in the green PCR (which used a relatively crude homogenate as the DNA template).

In order to detect knockout lines that contained more than one copy of the targeting construct, these lines were also screened by PCR using construct-specific, outward-pointing primers to detect concatenation events This is the lower central panel in Supplementary Figure 2b, and the source image is the lower panel in gel image yk180727-3.

This indicated that most of the KO transformants contained multiple insertions (which doesn't really matter, if they are located within the ERD2B-2 gene, because they are still gene knockouts, but it does matter if this PCR is detecting additional off-target insertions that are typically concatenated copies. Ideally, we want KO lines that (i) contain only single-copy gene knockouts and (b) contain no off-target events, as these might disrupt other genes and generate misleading phenotypes.

Only 4 lines, T351/3-15, T351/3-30, T351/3-32 and T351/3-41 contained no concatenated DNA. These lines were then re-tested to confirm that only a single copy of the targeting construct had replaced the ERD2B-2 gene, by using external locus-specific primers to amplify the targeted gene: this is the right-hand image in Supplementary Figure 2 b, and the source gel is yk180731-1.

All 4 lines passed this test and were additionally analysed by Southern blotting to confirm that they were "clean" KO lines (Supplementary Figure S2c) The source gel image is named 2018-05-23 gel and the corresponding blot hybridisation is named 2018-05-25 hyb. Note that this blot also shows the analysis of a set of lines that are unrelated to this project, and that were thus cropped from the image provided for this MS.

The four lines all contained the kanamycin/G418 resistance cassette, flanked by loxP sites. replacing the PpERD2B-2 gene. We selected one of these lines (line T351/3-15) for deletion of the selection cassette by cre-lox recombination. Following introduction of a transiently expressed 35S-Cre construct into protoplasts, regenerants were analysed for the presence/absence of the selection cassette by analysis of G418 sensitivity and PCR amplification with selection cassette specific PCR primers (Supplementary figure 2d) The upper panel shows retention of a selection cassette in line 7 of 13 lines tested, while amplification with primers flanking the site of the selection cassette confirmed its successful deletion in the remaining 12 lines. The source images are gel images yk181213-1 (central set of tracks) and yk181213-2 (central set of tracks). Note that both these gel images include analysis of other marker removal events from lines unrelated to this paper.

A note on multiple lines *versus* single genotyped lines.

It is common practice within the plant sciences community to generate multiple independent transgenic lines that are used for subsequent phenotypic analysis. The rationale for this (as a requirement in most journals) is that in angiosperms it has not been possible to undertake precision modification of specific loci, since the methods of transformation used (typically *Agrobacterium*-mediated DNA delivery) coupled with the propensity for the integration of DNA into genomes via the non-homologous end-joining pathway of DNA double-strand break repair typically results in efficient integration of transgenes at essentially random loci. Thus analysis of multiple transgenic events is required to ensure reproducibility.

This is **NOT** the case in *P. patens*, in which the targeted integration of transgenes with sequence identity with an endogenous locus typically results in high frequency of “gene targeting”. Once the accuracy of gene targeting (integration of a transgene at a specific locus, as a single copy with no associated off-target events) has been established, there is no necessity to generate multiple individual lines for phenotypic analysis, since all such lines are genetically identical.

It can easily be appreciated that where multiple independent loci are targeted, through an initially targeted line being subsequently re-transformed with a second construct, that a requirement to re-transform more than one successfully targeted mutant line would very substantially increase the workload required, to beyond what is practically possible. This is particularly the case where one of these lines needs to be delivered without a selection marker to enhance the efficiency of identifying transformants. This was the case in this instance where the initial construction of a precise knock-in into the ERD2B-1 gene required the manual genotyping of a very large pool of regenerating plants before the much simpler knockout of the ERD2B-2 gene could be undertaken.

Supplementary Figure 3

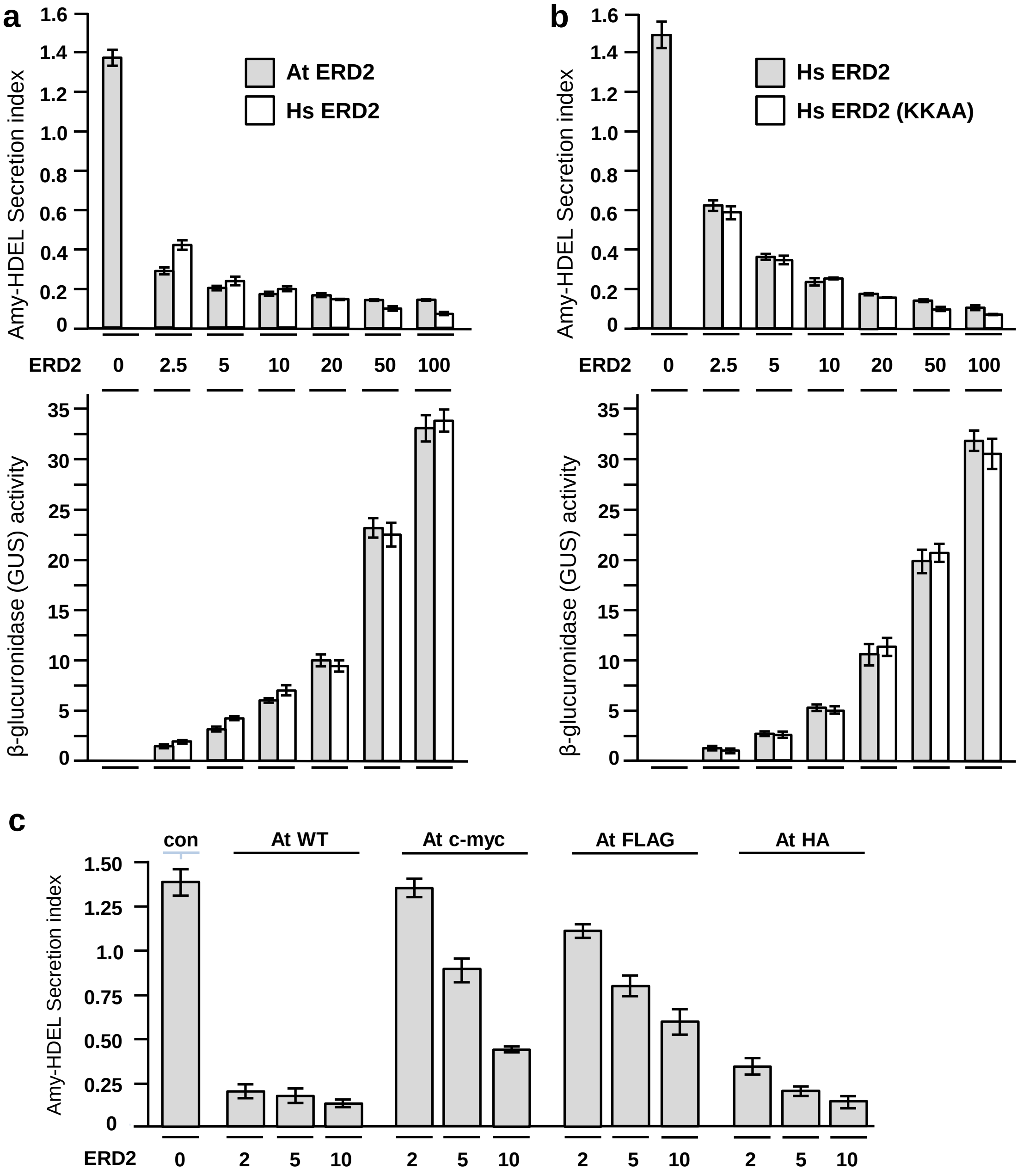

Supplemental Figure 3: Dose response comparisons between ERD2 derivatives.

a Transient expression experiments in which the reduction of the secretion index is a measure of ERD2 function (upper panel). A constant amount of cargo plasmid encoding Amy-HDEL is co-transfected with different amounts of effector plasmids (given as a percentage of the maximum dose in the series) encoding either At or Hs ERD2. The dose-response shows a lower retention efficiency of HsERD2 at low dosage, and a higher efficiency at high dosage, compared to AtERD2. Notice also that transfection efficiency is almost identical in the two cases as indicated by the internal marker GUS (lower panel), given in standard OD units. Error bars are standard deviations from 2 biological replicas.

b A dose-response assay as in (a), but comparing human ERD2 and the human ERD2 mutant KKAA. Notice the highly similar dose-response suggesting the silent nature of the double point mutation. Error bars are standard deviations from 2 biological replicas.

c Dose-response with the AtERD2 constructs from Fig. 3g. Notice that addition of c-myc and FLAG tags strongly inhibits ERD2 activity, whilst HA-tagging had only a small effect. Notice also that a dose-response is better suited to display the effect of epitope tagging compared to overexpression. Error bars are standard deviations from 3 biological replicas.

Source data are provided as a Source Data file.

Supplementary Figure 4

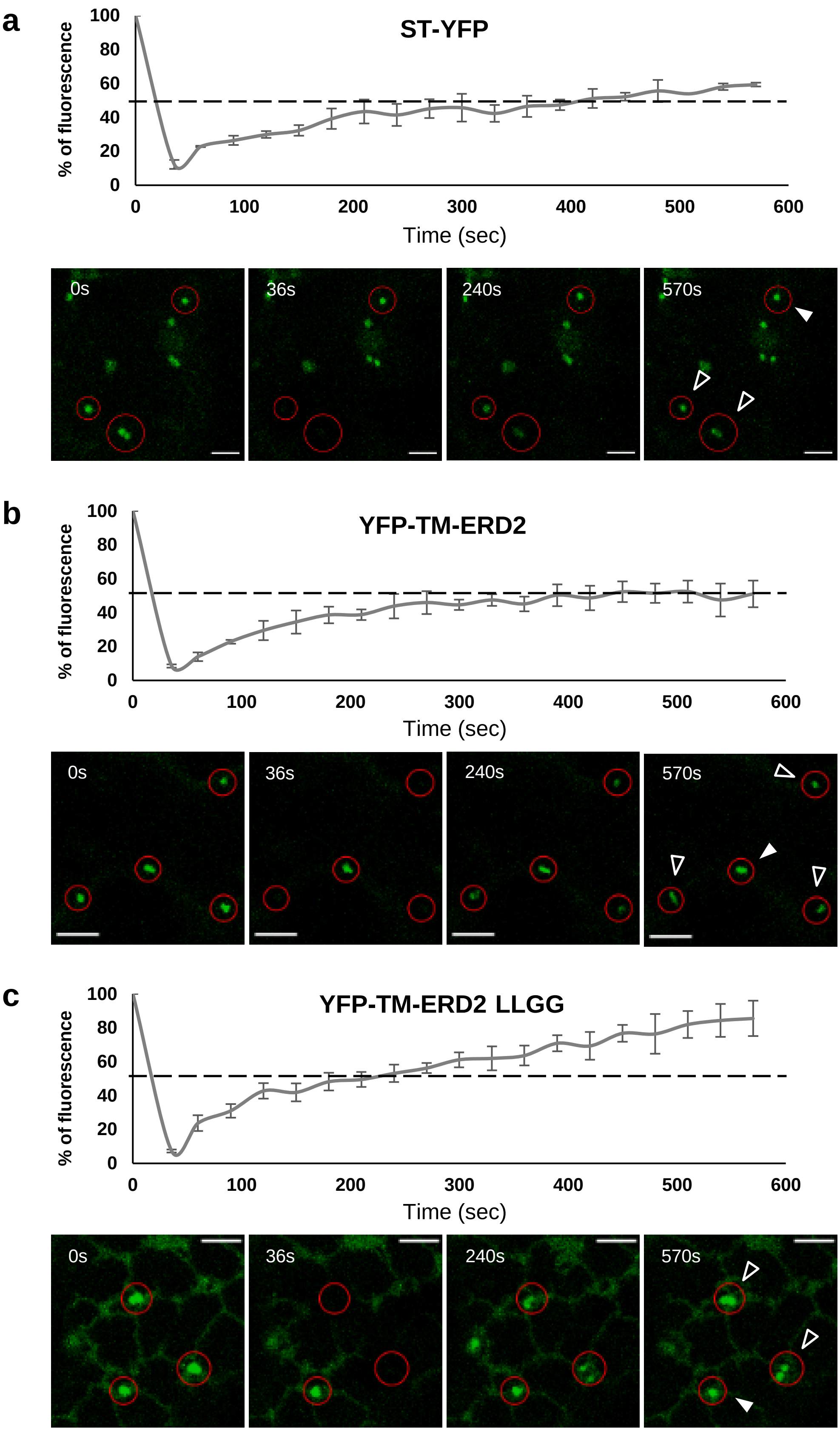

Supplemental Figure 4: Fluorescent recovery after photobleaching to characterise the di-leucine motif

Imaging data used to determine recovery rate for fluorescent constructs. Recordings were made from 5 independent video captures and 2-3 bleached Golgi bodies each time. The steady state fluorescence of the Golgi bodies before photobleaching was set to 100% each time in order to generate recovery percentages with error bars at each sampling time point. Four time points of a single video captures are provided for visual guidance. Bleached Golgi bodies recovering are indicated by open arrowheads, a non-bleached Golgi body is indicated by a closed arrow head. All size bars are 5 microns

a FRAP and Golgi-body tracking with ST-RFP to determine the baseline recovery rate of co-expressed ST-YFP, which was 50% after 400 seconds following photobleaching. Error bars are standard deviations from at least 3 biological replicas. Source data are provided as a Source Data file.

b&c Golgi-body tracking with ST-RFP to determine fluorescence recovery of co-expressed YFP-TM-ERD2 and the LLGG mutant. ERD2 recovery to 50% (400 seconds) took almost twice the time of the LLGG mutant (240 seconds). LLGG mutant recovery reached 85%, whereas wild type ERD2 remained at around 50%. Notice the presence of an ER network in the case of the LLGG mutant and how bleached Golgi bodies recovered close to the original fluorescence levels. Error bars are standard deviations from at least 2 biological replicas. Source data are provided as a Source Data file.

Supplementary Figure 5

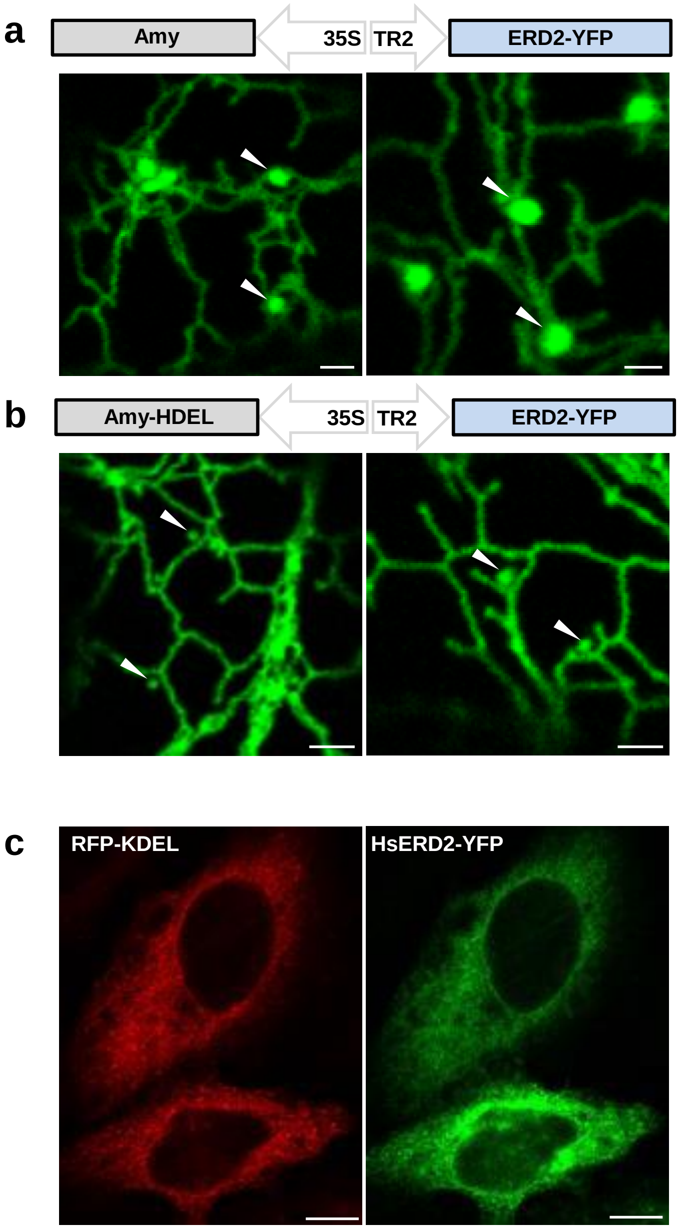

Supplemental Figure 5: C-terminally tagged ERD2 can be redirected to the ER by ligand overdose.

a ERD2-YFP co-expressed with control cargo Amy in leaf epidermis cells. Golgi-bodies with saturating levels of YFP fluorescence are indicated by white arrow heads. Size bar 2 microns.

b As in a) but ERD2-YFP was co-expressed with Amy-HDEL. Notice that although ER fluorescence is slightly stronger than in panel a, Golgi fluorescence intensity is no longer saturating and comparable to the ER fluorescence. Size bar 2 microns.

c HeLa cells co-expressing RFP-KDEL and YFP-TM-HsERD2. Notice that stronger expression of YFP-TM-HsERD2 leads to a stronger fluorescence at the Golgi region, whilst ER fluorescence is not much altered. Size bars 10 microns

Supplementary Figure 6

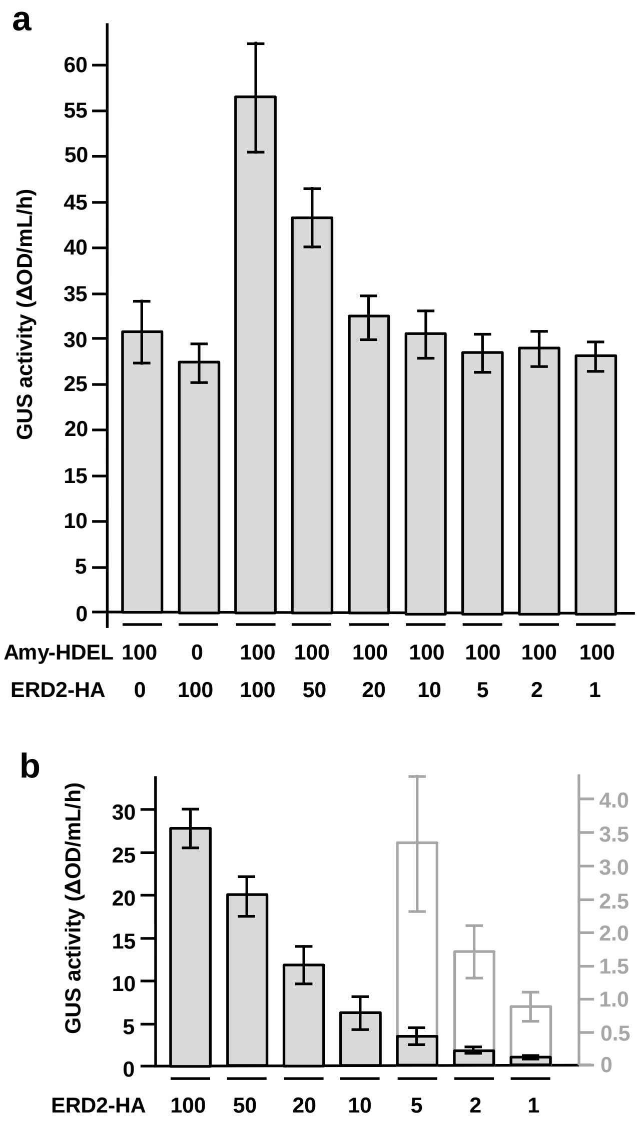

Supplemental Figure 6: Illustration of dose-response conditions

a. Measurement of GUS activities from protoplasts transfected with the maximum amount of dual expression vectors as deployed in Fig. 7a, transfected individually (first two lanes) and in combination, as well as all ERD2-HA plasmid dilutions from Fig. 7c. Notice that GUS levels from the individual plasmids are comparable, and that differences in the synthesis rate of ERD2-HA and Amy-HDEL observed in Fig. 7a, b are properties of the proteins themselves. Error bars are standard deviations from 10 biological replicas. Source data are provided as a Source Data file.

b To appreciate the quantitative nature of the GUS assay, the same dilution series of the ERD2-HA plasmid was transfected alone. GUS activities of the three highest dilutions are also shown at a 10-fold more sensitive scale (y-axis on the right). Error bars are standard deviations from 4 biological replicas. Source data are provided as a Source Data file.

Supplementary Table 1 – plasmids used in this study:

Description	Reference	Figure	name
TR2:ST-YFP-HDEL-35S:NbERD2abAS	J. Alvim	Figure 1	pTJCA85
TR2:YFP-1TM-ERD2b-35S:NbERD2abAS	J. Alvim	Figure 1	pTJCA86
35s:α-amylase-HDEL	(Phillipson et al. 2001)	Figures 2, 3, 4, 5, 6	pAmy-HDEL
35s:α-amylase-KDEL	(Silva-Alvim et al., 2018)	Figure 2	pAmy-KDEL
TR2:GUS 35s:ERD2b	(Silva-Alvim et al., 2018)	Figures 2, 4	pJA31
TR2:GUS 35s:oiERD2	Gene synthesis	Figure 2	pJCA3
TR2:GUS 35s:acERD2	Gene synthesis	Figure 2	pJCA68
TR2:GUS 35s:piERD2	Gene synthesis	Figure 2	pJCA21
TR2:GUS 35s:ccERD2	Gene synthesis	Figure 2	pJCA67
TR2:GUS 35s:gsERD2	Gene synthesis	Figure 2	pJCA66
TR2:GUS 35s:hERD2	Gene synthesis	Figure 2	pJCA4
TR2:GUS 35s:taERD2	Gene synthesis	Figure 2	pJCA70
TR2:GUS 35s:tpERD2	Gene synthesis	Figure 2	pJCA18
TR2:GUS 35s:pgERD2	Gene synthesis	Figure 2	pJCA22
TR2:GUS 35s:klERD2	Gene synthesis	Figure 2	pJCA63
TR2:GUS 35s:tbERD2	Gene synthesis	Figure 2	pJCA69
TR2:GUS 35s:yERD2	Gene synthesis	Figure 2	pJCA2
CMV:YFP-TM-AtERD2b	J. Alvim	Figure 2	pJCA108
CMV:AtERD2b-YFP	J. Alvim	Figure 2	pJCA109
CMV:HsERD2-YFP	R. Bolt	Figure 2	pRB52
TR2:YFP-1TM-hERD2	J. Alvim	Figure 3	pTJCA107
TR2:hERD2-YFP	R. Bolt	Figure 3	pTRB21
TR2:GUS 35S:hERD2	J. Alvim	Figure 3	pJCA4
TR2:YFP-1TM-hERD2 S209A	R. Bolt	Figure 3	pTRB14
TR2:YFP-1TM-hERD2 S209D	R. Bolt	Figure 3	pTRB15
TR2:YFP-1TM-hERD2 L208G^L210G	R. Bolt	Figure 3	pTRB16
TR2:GUS 35s:hERD2 S209A	J. Ranger	Figure 3	pJAR1
TR2:GUS 35s:hERD2 S209D	J. Ranger	Figure 3	pJAR2
TR2:GUS 35s:hERD2 L208G^L210G	J. Ranger	Figure 3	pJAR3
TR2:GUS 35s:hERD2 K206A	N. Bencka	Figure 3	pNB1
TR2:GUS 35s:hERD2 K207A	N. Bencka	Figure 3	pNB2
TR2:GUS 35s:hERD2 K206A^K207A	N. Bencka	Figure 3	pNB3
TR2:YFP-TM-ERD2 L211G^L213G	(Silva-Alvim et al. 2018)	Figure 3	pTJCA49
TR2:YFP-TM-ERD2ΔC5	J. Alvim	Figure 3	pTJCA88
TR2:ST-RFP	(Silva-Alvim et al. 2018)	Figure 3	pTJA37
TR2:GUS 35s:ERD2b-HA	J. An	Figures 3,7	pJA33
TR2:GUS 35s:ERD2b-FLAG	J.Denecke	Figure 3	pJD6
TR2:GUS 35s:hERD2-myc	D. Hirsz	Figure 3	pDH2
TR2:GUS 35s:hERD2-HA	D. Hirsz	Figure 3	pDH1
TR2:GUS 35s:hERD2-FLAG	D. Hirsz	Figure 3	pDH3

Supplementary Table 1 – plasmids used in this study: (continued)

Description	Reference	Figure	name
TR2:YFP-TM-ERD2-myc	D. Hirsz	Figure 3	pTDH2
TR2:YFP-TM-ERD2-HA	D. Hirsz	Figure 3	pTDH1
TR2:YFP-TM-ERD2-FLAG	D. Hirsz	Figure 3	pTDH3
35S:Amy TR2:YFP-TM-ERD2ΔC5	R. Bolt	Figure 4	pTRB6
35S:AmyHDEL TR2:YFP-TM-ERD2ΔC5	R. Bolt	Figure 4	pTRB7
35S:Amy TR2:YFP-TM-ERD2	R. Bolt	Figure 4	pTRB8
35S:AmyHDEL TR2:YFP-TM-ERD2	R. Bolt	Figure 4	pTRB9
35S:Amy TR2:ERD2b-YFP	M. Hu	Figure 4	pTMY3
35S:AmyHDEL TR2:ERD2b-YFP	M. Hu	Figure 4	pTMY4
TR2:ERD2b-YFP	M. Hu	Figure 4	pTMY1
TR2:YFP-TM-ERD2	(Silva-Alvim et al. 2018)	Figures 4,6	pTFLA32
TR2:GUS 35s:ERD2b::p24tail	J. Alvim	Figure 5	pJCA75
TR2:GUS 35s:ERD2b::p24tail KKSS	R. Bolt	Figure 5	pRB1
TR2:RFP-TM-ERD2::p24tail	J. Alvim	Figure 5	pTJCA76
TR2:RFP-TM-ERD2::p24tail KKSS	J. Alvim	Figure 5	pTJCA108
TR2:LPYS-TM-ERD2-YFP	R. Bolt	Figure 5	pTRB25
TR2:ST-YFP	(Bottanelli et al. 2012)	Figure 5	pST-YFP
TR2:RFP-TM-ERD2	F. Silva-Alvim	Figure 5	pTFLA41
TR2:ARF1-RFP	P. Manseau	Figure 5	pTPM3
TR2:GUS 35s: LPYS-TM-ERD2-YFP	R. Bolt	Figure 6	pRB25
TR2:GUS 35s: TM-ERD2-YFP	R. Bolt	Figure 6	pRB26
TR2:TM-ERD2-YFP	R. Bolt	Figure 6	pTRB26
TR2:NbMNS3-RFP	R. Bolt	Figure 6	pTRB23
TR2: LPYS-TM-ERD2-YFP	R. Bolt	Figure 6	pTRB25
TR2: TM-ERD2-YFP	R. Bolt	Figure 6	pTRB26
TR2:GUS 35s:AmyHDEL	J.An	Figure 7	pGAmyHDEL

Abbreviations: 35S: Cauliflower mosaic virus promoter; TR2': TR-DNA derived mas 2'; GUS: β-glucuronidase; Amy: Barley α-amylase; CMV: Cytomegalovirus

Supplementary Table 2 – Primer list:

All primers are written from 5’ to 3’ by convention

P1	ERDB2-1_S1	GATACGGAATTTGAGGTGCACAA
P2	ERDB2-1_OLA	CTCCTCGCCCTTGCTCACCATCCTGGCAGCTTCGTATACCCAG
P3	ERDB2-1_OLS	CACGATCACGACGAGATCTCAATGAATATCTTCAGGCTTACGGG
P4	ERDB2-1_A1	ACTGTCTTGTGGCGTCTCATGTA
P5	ERDB2-1_KIS	CCATGCAAAGGAGGTGTCTGATA
P6	ERDB2-1_KIA	TGCCCAGGAATATCAGCTTCAT
P7	S65T-140A	AACTTCAGGGTCAGCTTGCCGTAG
P8	S65T-631S	AGCAAAGACCCCAACGAGAAGC
P9	ERD2B-2_S1	AGATGGTTGGGGTTAGCATTTGT
P10	ERD2B-2_A1	TACCATGTCTGAAGAGCAAAGCA
P11	ERD2B-2_S2	GCAATGGTCTTTGTCTTCGGAAT
P12	ERD2B-2_A2	TGAATGCTGTTTCAAGCCTTACA
P13	ERD2B-2KOS	TGAGGGCAATTTTGAGAACAAAT
P14	ERD2B-2KOA	TCATGAATCCCAAATACCAATGA
P15	ERD2B-2_S3	ATGCCTTGACACCTGTAGCTGAG
P16	ERD2B-2_A3	TCACCCATTAGACTGGGAACAGA
P17	35Spro2R	AGATAGCTGGGCAATGGAATCCGA
P18	g6term4F	TAGGGTTCTTATAGGGTTTCGCTCA
P19	npt87S	TGACTGGGCACAACAGACAATC
P20	npt780A	GTCAAGAAGGCGATAGAAGGCGATG
P21	35SproXbaI	AGAGAGATCTAGACCCCTACTCCAAAAATGTCAAA

To amplify ERD2 sequences from small vectors for further subcloning

cool35S
CACTATCCTTCGCAAGACC

Human ERD2 fluorescent fusions

BglII-hERD2
GAGATCTCAATGAACATTTTCCGGCTG
huERD2-NheI
CTCATTGCGCTAGCTGCTGGCAAACTGAGCTTCTTTCCC

Plant ERD2 epitope tagging

FlagCter
TCCTGGTCTCTAGATTACTTGTCATCGTCATCCTTGTAATCAGCTGGTAATTGGAGCTTTTTGTTG
MycCter
TCCTGGTCTCTAGAGGTCCTCCTCGGAGATCAGCTTCTGCTCAGCTGGTAATTGGAGCTTTTTGTTG
HACter
TCCTGGTCTCTAGAAATTAAGCGTAATCTGGAACATCGTATGGGTAAGCTGGTAATTGGAGCTTTTTGTTG

Supplementary Table 2 – Primer list: (continued)

Human ERD2 epitope tagging

Rob flag
TCCTGGTCTCTAGATTACTTGTCATCGTCATCCTTGTAATCTGCTGGCAAACTGAGCTTCTTTCCC
Rob myc
TCCTGGTCTCTAGAGGTCCTCCTCGGAGATCAGCTTCTGCTCTGCTGGCAAACTGAGCTTCTTTCCC
Rob HA
TCCTGGTCTCTAGATTAAGCGTAATCTGGAACATCGTATGGGTATGCTGGCAAACTGAGCTTCTTTCCC

Human ERD2 mutagenesis

S209A sense AAAGTACTCAAGGGAAAGAAGCTCGCTTTGCCAGCATAATCTAGAGGA
S209A anti TCCTCTAGATTATGCTGGCAAAGCGAGCTTCTTTCCCTTGAGTACTTT

S209D sense AAAGTACTCAAGGGAAAGAAGCTCGATTTGCCAGCATAATCTAGAGGA
S209D anti TCCTCTAGATTATGCTGGCAAATCGAGCTTCTTTCCCTTGAGTACTTT

K206A sense ACAAAAGTACTCAAGGGAGCTAAGCTCAGTTTGCCAGCA
K206A anti TGCTGGCAAACTGAGCTTAGCTCCCTTGAGTACTTTTGT

K207A sense AAAGTACTCAAGGGAAAGGCTCTCAGTTTGCCAGCATAA
K207A anti TTATGCTGGCAAACTGAGAGCCTTTCCCTTGAGTACTTT

KKAA sense ATTACAAAAGTACTCAAGGGAGCTGCTCTCAGTTTGCCAGCATAATCT
KKAA anti AGATTATGCTGGCAAACTGAGAGCAGCTCCCTTGAGTACTTTTGTAAT

L208G^L210G anti CTTCGGATCCTCTAGATTATGCTGGACCACTTCCCTTCTTTCCCTTGAGTACTTTTGTA
L208G^L210G sense TACAAAAGTACTCAAGGGAAAGAAGGGAAGTGGTCCAGCATAATCTAGAGGATCCGAAG

Modifications to Arabidopsis thaliana ERD2

ERD2bΔC5 anti TAAGCTGGTCTAGATTACTTTTTGTTGTTCTTCCAGCTTAGGAAA

ERD2b::p24tail
TCTGCTTCGGATCCTCTAGATAAGTTTCTTCTTGTGGAAATACCTCTTCCAGCTTAGGAAATAATAATAGA

ERD2b::P24a KK^SS sense
TGGAAGAGGTATTTCCACAAGTCTTCTCTTATCTAGAGGATCCGAAG
ERD2b::P24a KK^SS anti
CTTCGGATCCTCTAGATAAGAGAAGACTTGTGGAAATACCTCTTCCA

LPYS sense
CATGTCTAATTCATTGCCTTACTCGGTGAAAGATGTGCATTATGATAACGCCAAGTTCCGCCAACGAT
LPYS anti
CGATCGTTGGCGGAACTTGGCGTTATCATAATGCACATCTTTCACCGAGTAAGGCAATGAATTAGA

Supplementary Table 2 – Primer list: (continued)

Cis-Golgi marker
MNS3 ClaI
CTTGATCTATCGATGTCGAATTCATTGCCTTACTCGGTG
MNS3 SalI
TGAAGTTGGTCGACCCATGAGTCGTGATATTGTCTCCTTC

Fluorescent ARF1 fusion
ARF1-NcoI
CACCAAATCCATGGGGTTGTCATTCGGAAAG
ARF1NheI
GCCATTGCGCTAGCTCCTGCCTTGCTTGCGATGTTGTTGG

Supplementary Table 3

Arabidopsis thaliana

MNIFRLAGDMTHLASVLVLLLKIHTIKSCAGVSLKTQELYAIVFATRYLDIFTSFVSLYNTSMKLVFLGSSFSIVWYMKYHK
AVHRTYDREQDTFRHWFLVLPCFLLALLIHEKFTFLEVLWTSSLYLEAVAILPQLVLLQRTRNIDNLTGQYIFLLGGYRGLYI
LNWIYRYFTEPHFVHWITWIAGFVQTLLYADFFYYYFLSWKNNKKLQLPA

Ostreococcus lucimarinus

MNIFRFAGDMTHLCSIVVLLLKIEATKSCAGVSLRTQELYAVVFVSRYLDLFFTFISVYNTVMKVFFITSSFCIIWYMRHHR
IVSQTYDREQDTFRVAFLVVPCIFLALLVNHEFSMVEVLWTFSIYLESVAILPQLILLQRTFNVDTLTSNYVFLLGAYRALYIL
NWLYRYFTEPGYSQWIVWSSGTLQTAIYCDFFYYYVVSWRKNERLSLPS

Chondrus crispus

MNIFRLGGDMLHVVSIFLLLLKIQTSHSCAGLSLKTQILYMVVFSTRYLDLFFTKPWHSALTFYNTIMKILFLSSSAYTIYL
MQKRYKHTYDKVHDTFRIQYLIAAAAVLALIFHLRLTVFEILWAFSVFLESVAILPQLFLLQETGEVENITSHYIFCLGGYRT
LYIFNWVWRYFTEHRRNQWLAWGCGTVQTLIYADFFYYYILSRKQGKKLRLPP

Galdieria sulphuraria

MNVFRIAGDLLHCISIFILLHKMRKTRTCTGISRKTLELYAIVFLTRYLDLFTGGYFDSALSLYNTVLKLLFLASTFYCVYLLRV
KYRHTYDRSHDTFRVPFLLGAAAVLAFIFPQRYTILEILWSFSQYLEAVAILPQLLLLQRTGEVENLTSHYIFCLGAYRGCYV
LNWIWRFFTDSTYRGQYVTWTAGLIQTSLYADFFYYYLKYKKQGRALKLPP

Homo sapiens

MNIFRLTGDLSHLAAIVILLLKIWKTRSCAGISGKSQLLFALVFTTRYLDLFTSFISLYNTSMKVIYLACSYATVYLIYLKFKAT
YDGNHDTFRVEFLVVPVGGLSFLVNHDFSPLEILWTFSIYLESVAILPQLFMISKTGEAETITTHYLFFLGLYRALYLVNWI
WRFYFEGFFDLIAVVAGVVQTILYCDFFYLYITKVLKGKKLSLPA

Hypsibius dujardini

MNIFRLAGDICHLAAIAVLLAKIWKTRSCAGISGKSQILFALVYTTRYVDLFFSFVSVYNSLMKAVFLIASFATVYLIYFKFK
ATYDFNHDTFRVEFLLIPCLILSLIITHSYEIVELLWTFSIYLEAVAILPQLFMVSKTGEAETITSHYLFALGAYRALYIANWIW
RFYAESFVDGIAVVAGIVQTILYADFFYLYITKVLKGKEFRLPA

Puccinia graminis

MNIFRLIGDLSHLASIFILIQKIIKSRSARGISFKTQVLYVVVFLTRYVDLVTGPFISIYNTAMKLFFIASSAYIVYLMHFKYKPT
QDPAIDTFKVEYLLGPCALLALVFNYKFTVVEVLWAFSIYLEAVAVFPQLFMLHRTGEAETITTHYLFALGLYRAMYIPN
WILRYTTENTLDPIAIFAGIVQTGLYADFFYIYFTRVMRGQKFELPA

Saccharomyces cerevisiae

MNPFRILGDLSHLTSILILIHNIKTTRYIEGISFKTQTLYALVFITRYLDLLTFHWVSLYNALMKIFFIVSTAYIVVLLQGSKRTN
TIAYNEMLMHDTFKIQHLLIGSALMSVFFHHKFTFLELAWSFSVWLESVAILPQLYMLSKGGKTRSLTVHYIFAMGLYRA
LYIPNWIWRYSTEDKKLDKIAFFAGLLQTLLYSDFFYIYYTKVIRGKGFKLPK

Kluyveromyces lactis

MLNVFRIAGDFSHLASIIILIQSITTSNSVDGISLKTQLLYTLVFITRYLNLFTKWTSLYNFLMKIVFISSSVYVIVLMRQQKFK
NPVAYQDMITRDQFKIKFLIVPCILLGLIFNYRFSFIQICWSFSLWLESVAILPQLFMLTKTGKAKQLTSHYIFALGLYRALYI
PNWIWRYYTEERFDKLSVFTGVIQTLVYSDFFYIYYQKVIKLGGDLELPQ

Supplementary Table 3 (continued)

Acanthamoeba castellanii
MNIFRIIGDLMHLSSILMLLWKIRATKSCAGVSLKTQEMYALVFVTRYLDIFWNFSSLYNSIMKIIFLGTSFAIIYFIRMKYR
HSYDKEHDSFRVVFLIGPALLLALVFNPEFSFFEILWAFSIYLEALAILPQLFLLQRTGEVETLTSHYIFALGGYRAFYLLNWI
YRLATEPGYSNWIVWIAGFVQTVLYMDFFYYYIQSKWYGKKFVLPA

Thalassiosira pseudonana
MNIFRLCGDMSHVFSIIVLLLRLRVARNAQGISLRTHELFLLVFLTRYTDLFTTYYSLYNSVMKVLYIASTASIVYTIRLQEPI
CSTYDKAQDTFRHWEFAVAPCAVLATLTHLISGGGLFSVVDVQELLWTFSIYLEAVAILPQLIVLQRYRDVENLTGNYIFF
MGLYRALYIVNWVFRAYNEPGYRHHYVVYFCGVLQTLLYADFFYYYVMSKRRGGKFSLPTKG

Phytophthora infestans
MNLFRLVGDMAHLASFLVLLLKLLASRSANGISLKSQELFFLVFVTRYVDLFFHFVSLYNTLMKLLFLLFSGAIVYVIRFKEP
FRSTYDKSHDAFLHIKFAVLPCALLALVFNEQFEVMEILWTFSIYLEAVAIIPQLILLQRHAEVENLTSNYVVLLGAYRGCYV
LNWIYRAATESSYHFIWLMFIAGMVQTALYVDFFYYYAISKYHGKKMTLPS

Uncropped gels for Supplementary Figure 1b

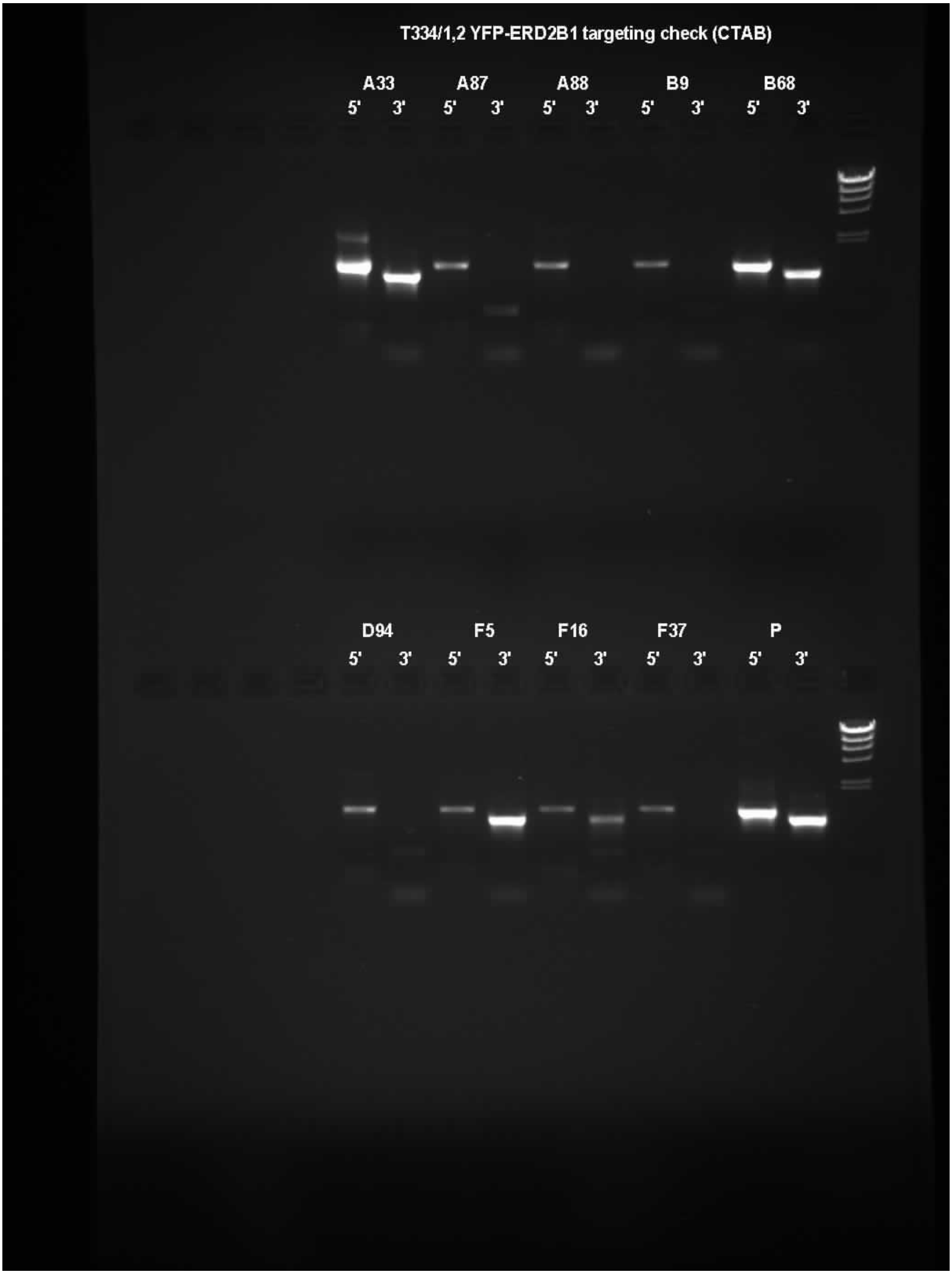

Uncropped gels for Supplementary Figure 1b (continued)

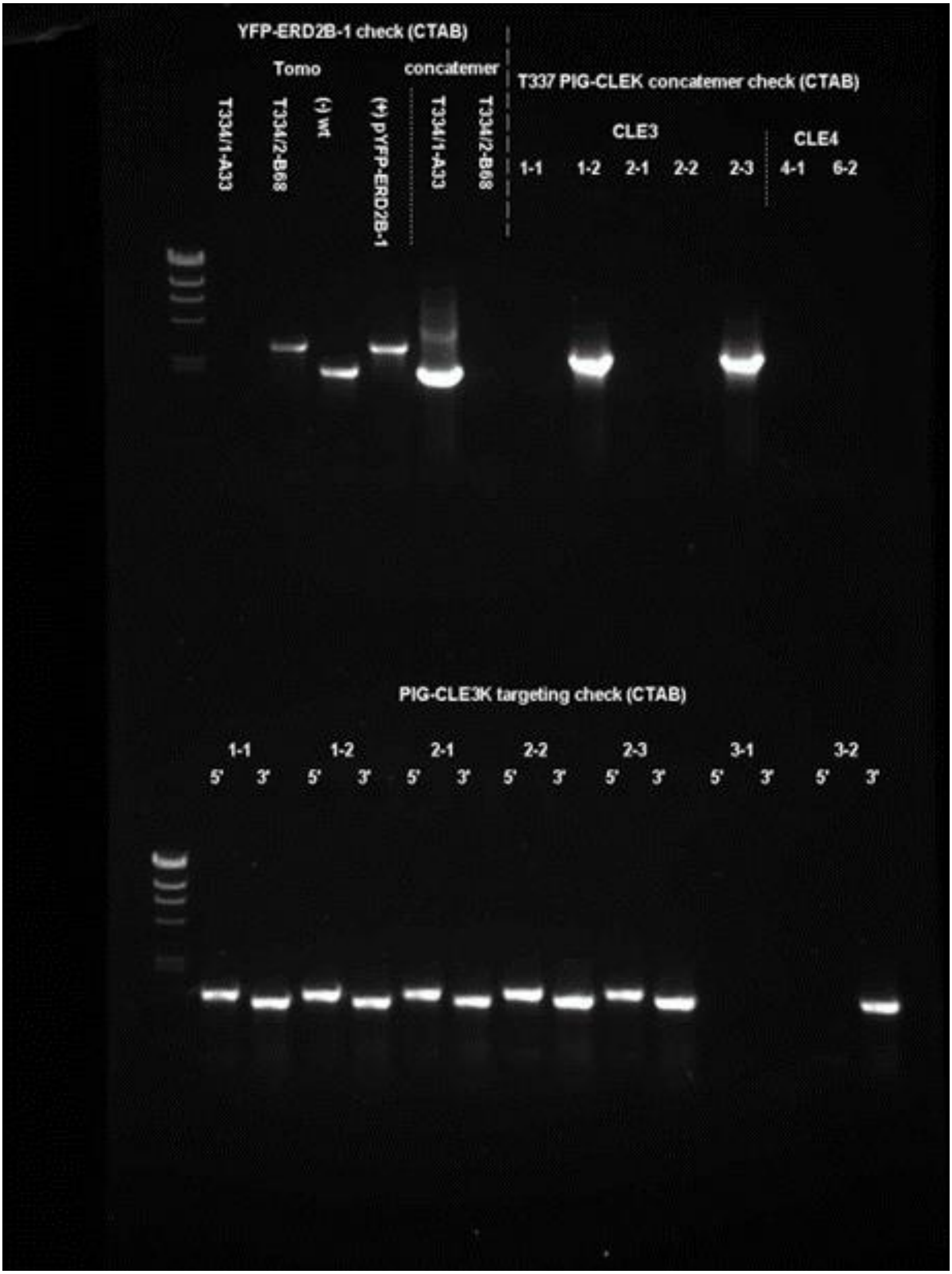

Uncropped gels for Supplementary Figure 1c

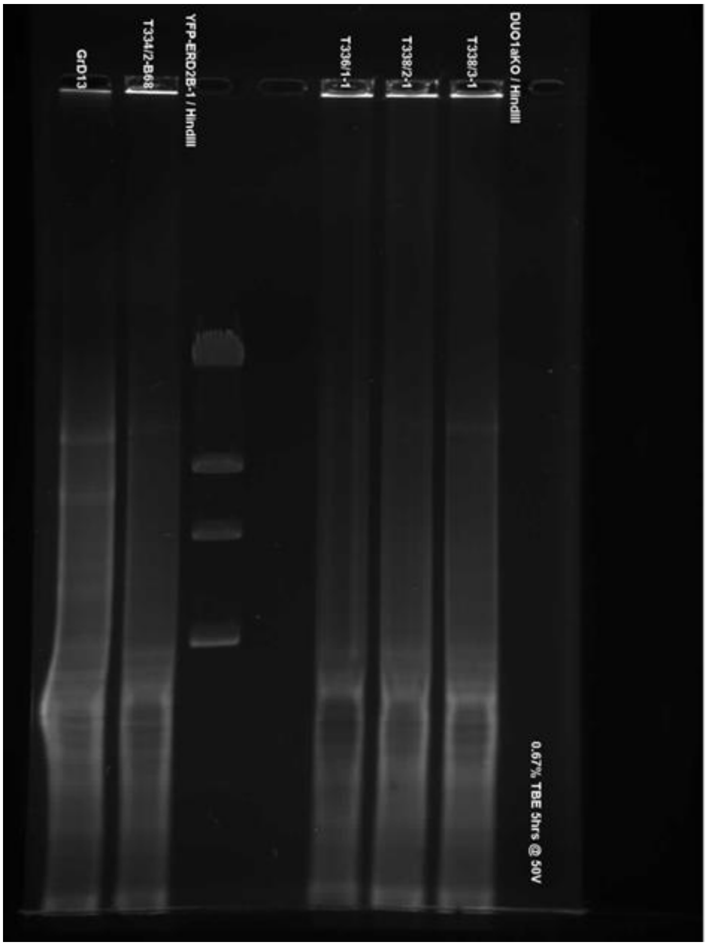

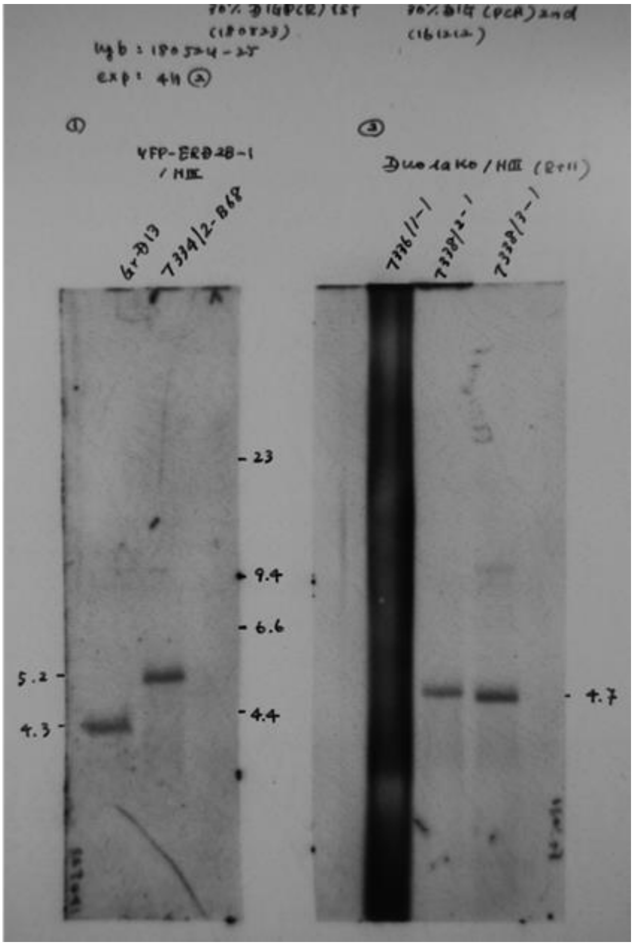

Uncropped gels for Supplementary Figure 2b

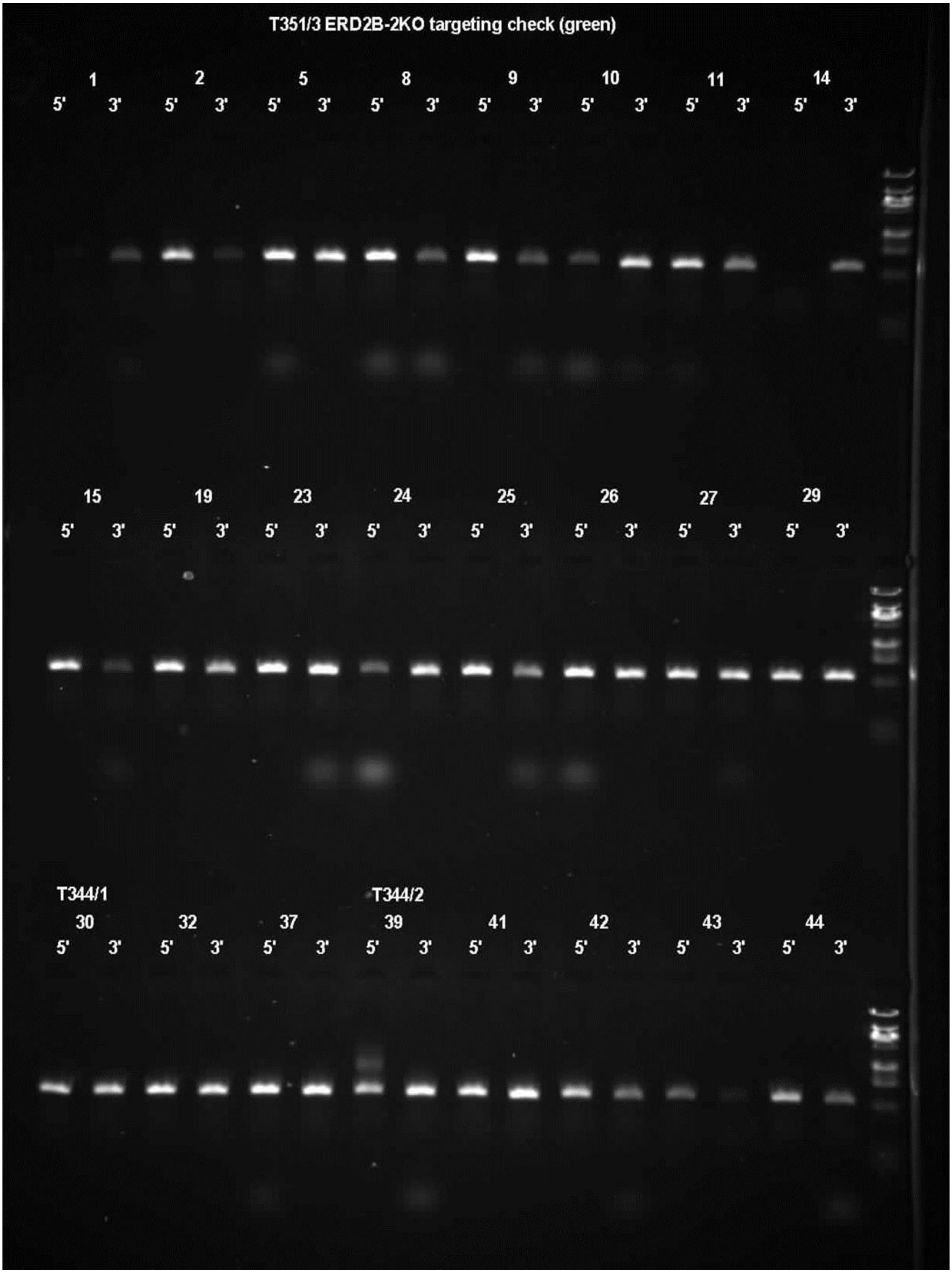

Uncropped gels for Supplementary Figure 2b (continued)

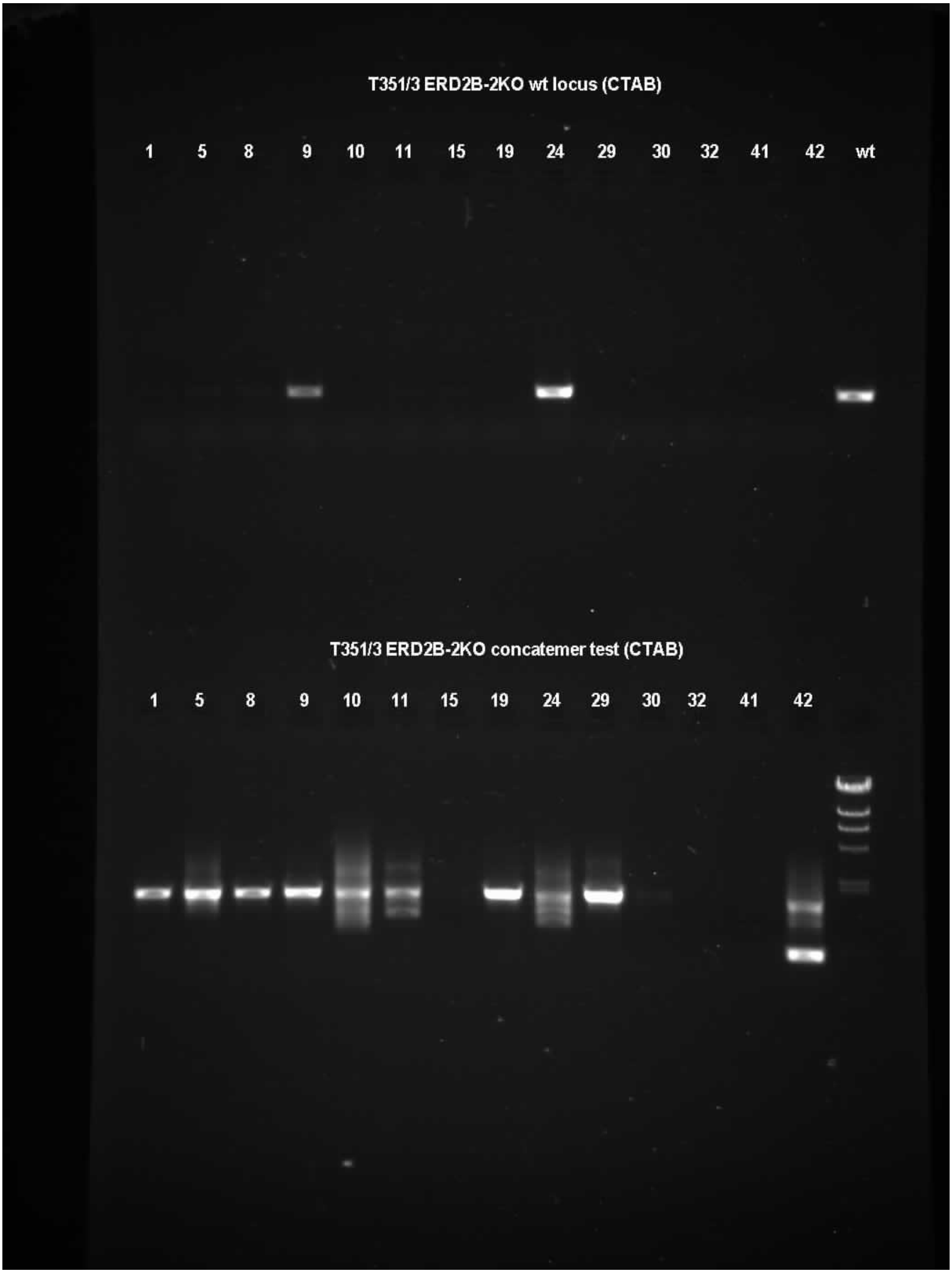

Uncropped gels for Supplementary Figure 2b (continued)

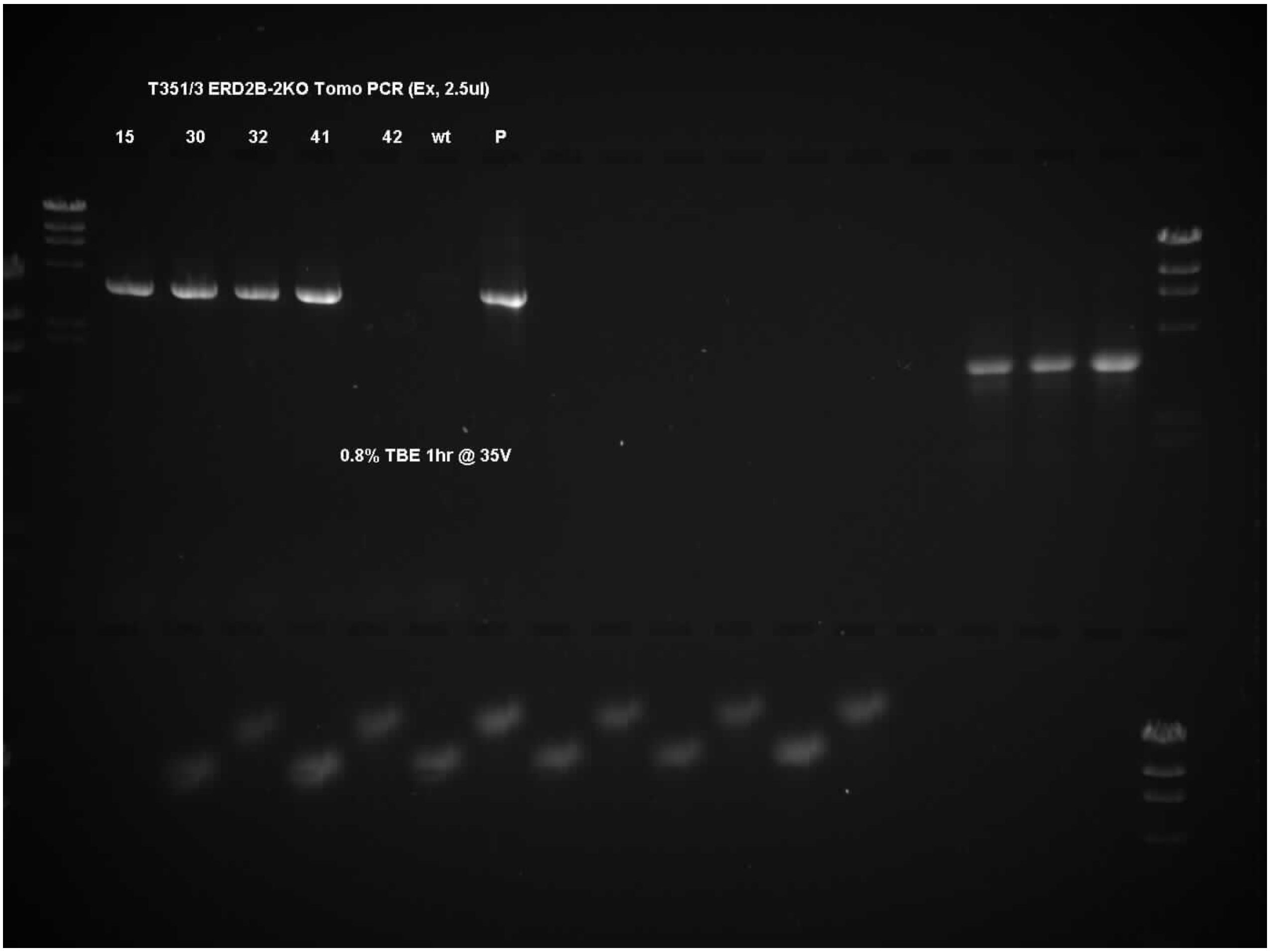

Uncropped gels for Supplementary Figure 2c

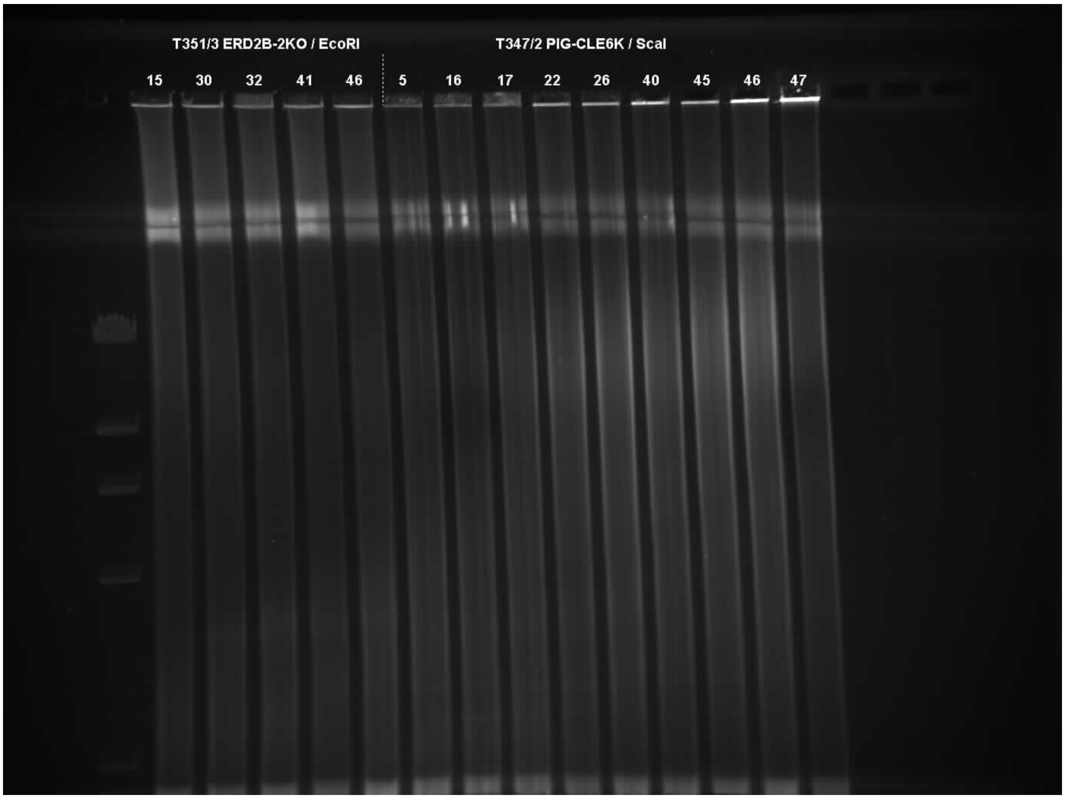

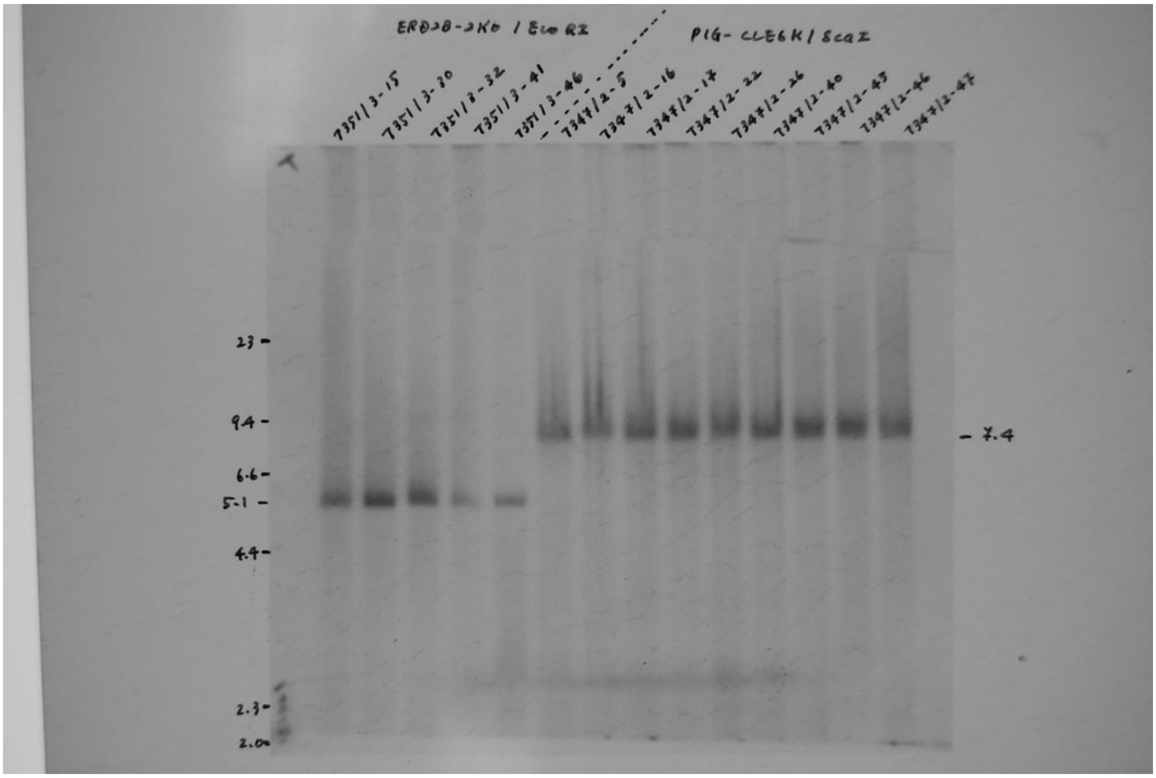

Uncropped gels for Supplementary Figure 2d

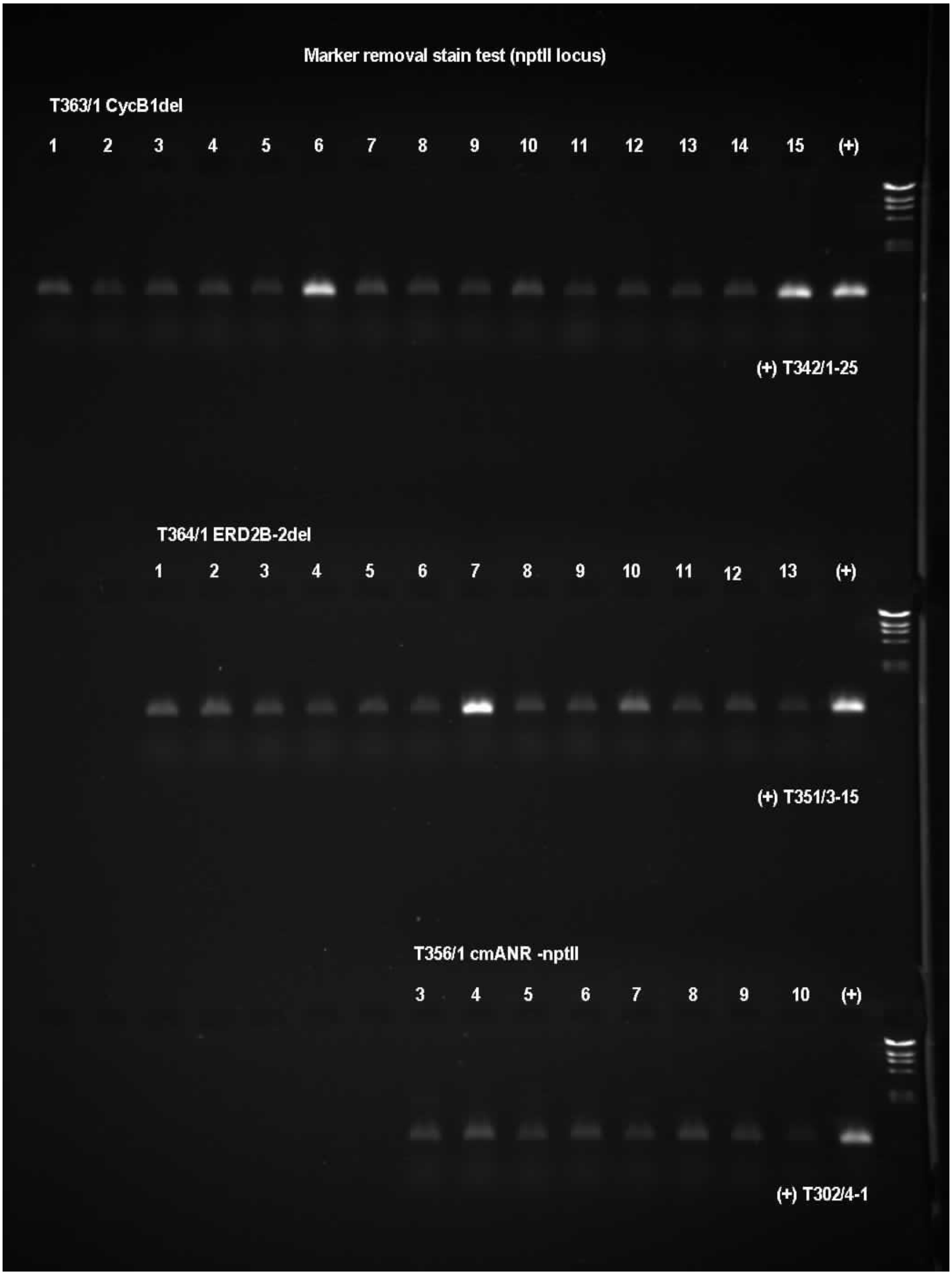

Uncropped gels for Supplementary Figure 2d (continued)

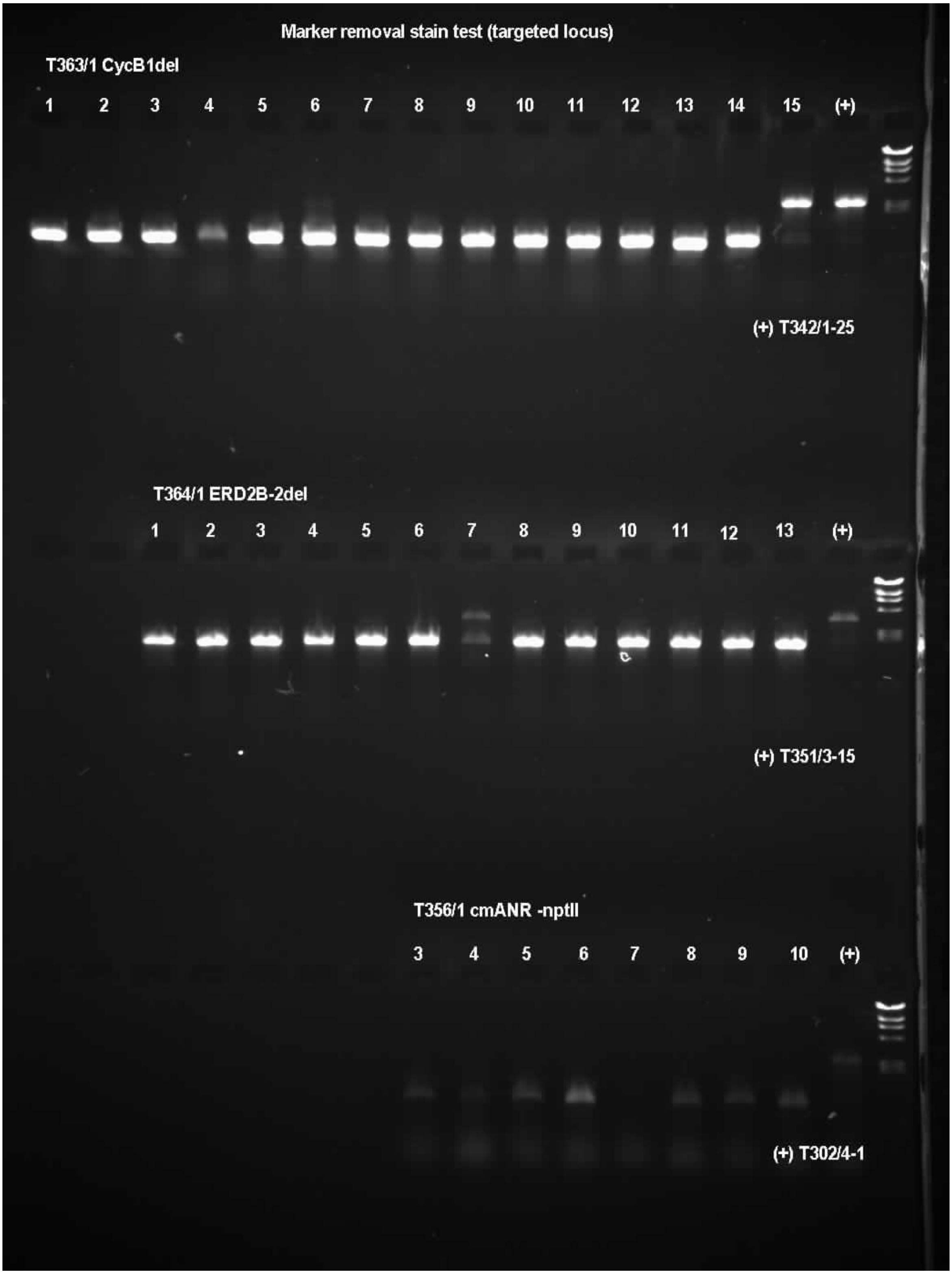

Supplement: Supplementary file 1 — Supplementary Information [file 41467_2023_37056_MOESM1_ESM.pdf]
