# Supplementary material for: The K/HDEL receptor does not recycle but instead acts as a Golgi-gatekeeper
Source: Nat Commun. 2023 Mar 23;14:1612. doi: 10.1038/s41467-023-37056-0 (PMC10036638; doi:10.1038/s41467-023-37056-0)
Supplement: Supplementary file 3 — Reporting Summary [file 41467_2023_37056_MOESM3_ESM.pdf]

nature portfolio

Corresponding author(s): Jurgen Denecke

Last updated by author(s): Feb 2, 2023

Reporting Summary

Nature Portfolio wishes to improve the reproducibility of the work that we publish. This form provides structure for consistency and transparency in reporting. For further information on Nature Portfolio policies, see our Editorial Policies and the Editorial Policy Checklist.

Statistics

For all statistical analyses, confirm that the following items are present in the figure legend, table legend, main text, or Methods section.

n/a	Confirmed	
☐	☒	The exact sample size (*n*) for each experimental group/condition, given as a discrete number and unit of measurement
☐	☒	A statement on whether measurements were taken from distinct samples or whether the same sample was measured repeatedly
☒	☐	The statistical test(s) used AND whether they are one- or two-sided<br>*Only common tests should be described solely by name; describe more complex techniques in the Methods section.*
☒	☐	A description of all covariates tested
☒	☐	A description of any assumptions or corrections, such as tests of normality and adjustment for multiple comparisons
☐	☒	A full description of the statistical parameters including central tendency (e.g. means) or other basic estimates (e.g. regression coefficient) AND variation (e.g. standard deviation) or associated estimates of uncertainty (e.g. confidence intervals)
☒	☐	For null hypothesis testing, the test statistic (e.g. *F*, *t*, *r*) with confidence intervals, effect sizes, degrees of freedom and *P* value noted<br>*Give P values as exact values whenever suitable.*
☒	☐	For Bayesian analysis, information on the choice of priors and Markov chain Monte Carlo settings
☒	☐	For hierarchical and complex designs, identification of the appropriate level for tests and full reporting of outcomes
☐	☒	Estimates of effect sizes (e.g. Cohen's *d*, Pearson's *r*), indicating how they were calculated

Our web collection on statistics for biologists contains articles on many of the points above.

Software and code

Policy information about availability of computer code

Data collection	Zen 2.3 black edition (Zeiss)
Data analysis	Zen 2.3 lite blue edition (Zeiss) and ImageJ ((http://rsb.info.gov/ij/)).Image analysis was undertaken using the ImageJ analysis programand the PSC co-localization plug-in

For manuscripts utilizing custom algorithms or software that are central to the research but not yet described in published literature, software must be made available to editors and reviewers. We strongly encourage code deposition in a community repository (e.g. GitHub). See the Nature Portfolio guidelines for submitting code & software for further information.

Data

Policy information about availability of data

All manuscripts must include a data availability statement. This statement should provide the following information, where applicable:

- Accession codes, unique identifiers, or web links for publicly available datasets
- A description of any restrictions on data availability
- For clinical datasets or third party data, please ensure that the statement adheres to our policy

This project has generated a large number of raw data that require standard manipulations such as subtracting a blank OD, calculating enzyme activities, calculating ratios between medium and cell samples, calculating averages, standard deviations and standard errors, all of which can be made available by the Lead contact upon reasonable request. For Figure 2A, the raw data for cells and medium, as well as the calculation of the secretion index can be found in the Source Data. Individual averages from separate biological replicas for Figures 3B, 3F, 5B, 5E, 6C, 7D and Supplementary Figures 3A, 3B, 3C, 6A, 6B, are included in Source Data. Uncropped gel images, blots, autoradiographs and FRAP data (Figures 1, 4A, 7 and Supplementary Figure 4A) can also be found in Source Data. Source data are provided with this paper.

Human research participants

Policy information about studies involving human research participants and Sex and Gender in Research.

Reporting on sex and gender	N/A
Population characteristics	N/A
Recruitment	N/A
Ethics oversight	N/A

Note that full information on the approval of the study protocol must also be provided in the manuscript.

Field-specific reporting

Please select the one below that is the best fit for your research. If you are not sure, read the appropriate sections before making your selection.

☒ Life sciences ☐ Behavioural & social sciences ☐ Ecological, evolutionary & environmental sciences

For a reference copy of the document with all sections, see nature.com/documents/nr-reporting-summary-flat.pdf

Life sciences study design

All studies must disclose on these points even when the disclosure is negative.

Sample size	Typical sample size was two to three million protoplasts in volumes of 2-2.5 mL cell suspensions for all transport experiments to minimise pipetting errors and artefacts such as protoplasts adhering to petri-dish surface.
Data exclusions	No data were excluded.
Replication	We use a combination of repeats with dose response assays and can illustrate reproducibility visually in our graphs. The source data can be consulted for further details.
Randomization	Not Applicable as the experiments were performed on millions of cells.
Blinding	Co-localisation studies were performed by a colour-blind investigator to avoid sample bias.

Behavioural & social sciences study design

All studies must disclose on these points even when the disclosure is negative.

Study description	N/A
Research sample	N/A
Sampling strategy	N/A

Data collection	N/A
Timing	N/A
Data exclusions	N/A
Non-participation	N/A
Randomization	N/A

Ecological, evolutionary & environmental sciences study design

All studies must disclose on these points even when the disclosure is negative.

Study description	N/A
Research sample	N/A
Sampling strategy	N/A
Data collection	N/A
Timing and spatial scale	N/A
Data exclusions	N/A
Reproducibility	N/A
Randomization	N/A
Blinding	N/A

Did the study involve field work? ☐ Yes ☒ No

Reporting for specific materials, systems and methods

We require information from authors about some types of materials, experimental systems and methods used in many studies. Here, indicate whether each material, system or method listed is relevant to your study. If you are not sure if a list item applies to your research, read the appropriate section before selecting a response.

Materials & experimental systems

n/a	Involved in the study	
☐	☒	Antibodies
☐	☒	Eukaryotic cell lines
☒	☐	Palaeontology and archaeology
☒	☐	Animals and other organisms
☒	☐	Clinical data
☒	☐	Dual use research of concern

Methods

n/a	Involved in the study	
☒	☐	ChIP-seq
☒	☐	Flow cytometry
☒	☐	MRI-based neuroimaging

Antibodies

Antibodies used	Anti-barley alpha amylase is a polyclonal anti serum from rabbit, a kind gift by Birte Svensson (Carlsberg Laboratory, Copenhagen, Denmark) over two decades ago. anti-HA epitope (cat # GTX11504, Genetex); anti-GM130 (cat # 610822; clone 35/GM130; BD Biosciences), anti-TGN46 (cat # AHP500; Bio-Rad),Donkey anti-mouse Alexa Fluor647 (Thermo cat # A3157-1) and Donkey anti-sheep Alexa Fluor594 (Thermo cat # A11016)
Validation	anti-barley alpha amylase is described in Phillipson et al. (2001) and cited.<br>All other antibodies are heavily commercialised, validated and readily available for purchase

Eukaryotic cell lines

Policy information about cell lines and Sex and Gender in Research

Cell line source(s)	HeLa CCL-2 cells were purchased from the American Type Culture Collection (Manassas, VA).
Authentication	Please refer to American Type Culture Collection website for further information.
Mycoplasma contamination	Negative tests confirmed.
Commonly misidentified lines<br>(See ICLAC register)	N/A
